# Statin Therapy and Cardiovascular Prevention: Contemporary Evidence, Challenges, and Future Directions—A Narrative Review

**DOI:** 10.3390/ijerph23070921

**Published:** 2026-07-17

**Authors:** Don Zachariah, Dominic Kakooza, Sharifa Pothas, Anjana Thomas, Lanthe Kruger

**Affiliations:** Faculty of Health Sciences, North West University, Potchefstroom 2520, South Africa

**Keywords:** statins, cardiovascular prevention, dyslipidaemia, medication adherence, statin-associated muscle symptoms, statin intolerance, precision medicine, behavioural medicine, pharmacogenomics

## Abstract

**Highlights:**

**Public health relevance—How does this work relate to a public health issue?**

- Cardiovascular disease remains the leading cause of mortality worldwide, and statin therapy is a cornerstone of cardiovascular prevention, with robust evidence supporting its role in reducing cardiovascular morbidity and mortality.
- Despite established efficacy, poor medication adherence, treatment discontinuation, statin-associated muscle symptoms, and negative perceptions of therapy continue to limit the population-level benefits of lipid-lowering treatment.

**Public health significance—Why is this work of significance to public health?**

- This review critically synthesises evidence on the biological, behavioural, psychological, social, and physiological factors that influence statin use and long-term treatment effectiveness.
- By examining the multifactorial nature of statin-associated muscle symptoms, medication adherence, physiological outcomes, and emerging precision medicine approaches, this review highlights areas of established evidence, ongoing controversy, and important knowledge gaps.

**Public health implications—What are the key implications or messages for practitioners, policy makers and/or researchers in public health?**

- Cardiovascular prevention strategies should prioritise patient education, shared decision-making, and interventions addressing medication beliefs, misinformation, nocebo effects, and barriers to long-term treatment adherence.
- Future research and policy should support multidimensional and equitable approaches to cardiovascular prevention while ensuring that emerging precision medicine strategies are clinically validated, accessible, and representative of diverse populations.

**Abstract:**

Cardiovascular disease (CVD) remains the leading cause of morbidity and mortality worldwide, and lowering low-density lipoprotein cholesterol (LDL-C) remains a cornerstone of cardiovascular prevention. Statins are among the most extensively studied and widely prescribed medications and have demonstrated substantial benefits in reducing major adverse cardiovascular events in both primary and secondary prevention settings. Nevertheless, the effectiveness of statin therapy in routine clinical practice is frequently compromised by poor adherence, treatment discontinuation, concerns regarding adverse effects, and persistent residual cardiovascular risk. This narrative review synthesises contemporary evidence relating to the mechanisms of action of statins, their role in primary and secondary prevention, determinants of medication adherence, statin-associated muscle symptoms (SAMSs), and emerging developments in precision cardiovascular medicine. Current evidence indicates that although statins remain highly effective in reducing cardiovascular risk, long-term treatment success is strongly influenced by behavioural, psychological, social, and healthcare system factors. Increasing attention has also been directed towards the multifactorial nature of SAMSs and the contribution of nocebo effects to perceived statin intolerance. Emerging approaches involving pharmacogenomics, artificial intelligence, digital health technologies, and multidimensional risk assessment offer opportunities for more individualised prevention strategies, although important limitations relating to cost, accessibility, and external validity remain. Overall, contemporary cardiovascular prevention requires a patient-centred approach that integrates biological, behavioural, and social determinants of health to optimise treatment adherence and improve long-term cardiovascular outcomes.

## 1. Introduction

Cardiovascular disease (CVD) continues to represent one of the greatest global public health challenges and remains the leading cause of death worldwide. Atherosclerotic cardiovascular disease (ASCVD), including coronary artery disease, myocardial infarction, peripheral arterial disease, and stroke, is strongly associated with elevated concentrations of low-density lipoprotein cholesterol (LDL-C), making lipid management a fundamental component of cardiovascular prevention [1,2].

Since their introduction, statins have revolutionised cardiovascular prevention and have become the cornerstone of lipid-lowering therapy because of their well-established efficacy in reducing LDL-C concentrations and preventing major cardiovascular events. Evidence from large randomised controlled trials and meta-analyses has consistently demonstrated that statins reduce cardiovascular morbidity and mortality in both primary and secondary prevention populations [3] (Cholesterol Treatment Trialists’ Collaboration, 2019). Consequently, international guidelines recommend intensive lipid-lowering strategies, particularly among individuals at high and extremely high cardiovascular risk [1,4].

Despite these advances, substantial challenges remain. Poor adherence and premature discontinuation of statin therapy are common and contribute significantly to residual cardiovascular risk. Concerns regarding adverse effects, particularly statin-associated muscle symptoms (SAMSs), negative medication beliefs, inadequate risk perception, and the influence of misinformation frequently undermine long-term treatment persistence. Moreover, emerging evidence suggests that behavioural, psychological, and social determinants of health may influence treatment initiation, adherence, and clinical outcomes to a greater extent than previously recognised [5].

The increasing emphasis on patient-centred care and precision medicine has expanded the scope of cardiovascular prevention beyond conventional lipid reduction. Advances in pharmacogenomics, artificial intelligence, digital health technologies, and multidimensional risk assessment have created opportunities for more personalised approaches to treatment. However, the translation of these innovations into routine clinical practice remains constrained by issues relating to cost-effectiveness, accessibility, validation, and health inequalities.

Although numerous reviews have examined individual aspects of statin therapy, few have attempted to integrate biological mechanisms, behavioural determinants, statin-associated muscle symptoms, social factors, and emerging precision medicine approaches within a single conceptual framework. Furthermore, considerable debate persists regarding the interpretation of statin intolerance, the contribution of nocebo effects, and the clinical utility of novel biomarkers and physiological outcomes.

Therefore, the aim of this narrative review is to critically synthesise contemporary evidence relating to the role of statins in cardiovascular prevention, with particular emphasis on medication adherence, statin-associated muscle symptoms, social determinants of health, and future developments in precision cardiovascular medicine. Areas of uncertainty, ongoing controversies, and priorities for future research are also discussed.

## 2. Review Methodology

### 2.1. Review Design

This study was conducted as a narrative review designed to provide a comprehensive synthesis of contemporary evidence relating to statin therapy and cardiovascular prevention. The review aimed to integrate evidence concerning mechanisms of action, clinical effectiveness, medication adherence, statin-associated muscle symptoms, muscle function, social determinants of health, and emerging precision medicine approaches.

### 2.2. Literature Search Strategy

A structured literature search was conducted using five electronic databases, namely PubMed/MEDLINE, Scopus, Web of Science, Embase, and the Cochrane Library, to identify relevant studies and guideline documents pertaining to statin therapy and cardiovascular prevention. The search included articles published between January 2015 and March 2026 to ensure the inclusion of contemporary evidence. In addition, manual searches of reference lists of eligible studies and relevant international clinical practice guidelines were performed to identify further publications that may not have been captured through the electronic database searches.

The search strategy employed combinations of keywords and Medical Subject Headings (MeSH) related to the major themes of the review. These included terms such as “statins,” “cardiovascular prevention,” “LDL cholesterol,” “medication adherence,” “statin persistence,” “statin-associated muscle symptoms,” “statin intolerance,” and “nocebo effect.” Additional search terms related to physiological outcomes and emerging approaches in cardiovascular prevention included “handgrip strength”, “skeletal muscle mass”, “phase angle”, “social determinants of health”, “precision medicine”, “pharmacogenomics”, “digital health”, “artificial intelligence”, “PCSK9 inhibitors”, “ezetimibe”, and “bempedoic acid”. Boolean operators (“AND” and “OR”) were used appropriately to combine search terms and optimise retrieval of relevant research. The complete electronic search strategy for PubMed is presented in Supplementary Materials Table S1.”

### 2.3. Eligibility Criteria

Priority was given to high-quality evidence, including international clinical practice guidelines, consensus statements, systematic reviews, meta-analyses, randomised controlled trials, prospective cohort studies, and observational studies with direct clinical relevance to cardiovascular prevention and statin therapy. Studies were selected based on their relevance to the objectives of the review and their contribution to understanding the biological, behavioural, physiological, and precision medicine dimensions of statin use.

Conference abstracts, editorials, case reports, and studies lacking adequate methodological information were excluded. Furthermore, only articles published in English were considered. Owing to the heterogeneity of study designs and outcomes across the included literature, findings were synthesised narratively rather than quantitatively, with greater emphasis placed on evidence derived from major clinical trials, contemporary guidelines, and systematic reviews.

After eliminating duplicates, screening of titles and abstracts was performed independently by two reviewers based on the objectives of this narrative review. Subsequently, full-text screening was performed independently by two reviewers. In case of any disagreement in determining eligibility or interpretation, resolution was achieved by consensus after discussion. Since this is a narrative review, inter-rater agreement measures were not computed.

The quality of evidence is judged by the study design and the robustness of the methodology. Highest preference was given to international evidence-based clinical guidelines, Cochrane systematic reviews, high-quality systematic reviews and meta-analysis, randomised controlled trials, and good-quality prospective cohort studies. In cases of conflicting evidence, higher importance was given to studies with low risk of bias, large sample size, long follow-up period, and multiple studies with consistent results.

### 2.4. Evidence Synthesis

Because of the heterogeneity of study designs, populations, and outcomes, a quantitative synthesis was not undertaken. Instead, evidence was synthesised narratively and organised into thematic sections. Greater emphasis was placed on findings from major clinical trials, contemporary guidelines, and systematic reviews. Particular attention was given to areas of controversy, limitations of current evidence, and emerging concepts relevant to patient-centred cardiovascular prevention.

## 3. Mechanisms of Action of Statins

Statins exert their lipid-lowering effects primarily through competitive inhibition of 3-hydroxy-3-methylglutaryl-coenzyme A (HMG-CoA) reductase, the rate-limiting enzyme in hepatic cholesterol synthesis. Reduced intracellular cholesterol concentrations stimulate the upregulation of hepatic low-density lipoprotein receptors, thereby enhancing the clearance of circulating LDL cholesterol (LDL-C) and reducing plasma LDL-C concentrations. The causal relationship between LDL-C reduction and atherosclerotic cardiovascular disease (ASCVD) prevention has been consistently demonstrated through genetic studies, epidemiological investigations, and randomised clinical trials [6].

Beyond lipid lowering, statins possess several pleiotropic properties that may contribute to cardiovascular protection. These include anti-inflammatory, antioxidant, endothelial-protective, and plaque-stabilising effects. Statins have been shown to reduce vascular inflammation, improve endothelial nitric oxide bioavailability, decrease oxidative stress, and promote stabilisation of vulnerable atherosclerotic plaques [7]. These mechanisms may partially explain the reduction in cardiovascular events observed in major statin trials.

However, the relative contribution of these pleiotropic effects remains controversial. Although experimental studies support anti-inflammatory and endothelial benefits, most evidence suggests that the majority of cardiovascular risk reduction is attributable to LDL-C lowering itself rather than independent pleiotropic mechanisms [6]. Consequently, the clinical importance of non-lipid effects should be interpreted cautiously.

Evidence from the Cholesterol Treatment Trialists’ Collaboration demonstrated that each 1 mmol/L reduction in LDL-C is associated with approximately a 20–25% reduction in major vascular events, irrespective of baseline cardiovascular risk [3]. These findings reinforce the concept that “lower is better” for LDL-C and provide the scientific basis for contemporary intensive lipid-lowering strategies.

## 4. Statins in Primary and Secondary Prevention

### 4.1. Primary Prevention

Primary prevention aims to reduce the occurrence of first cardiovascular events among individuals without established ASCVD. Numerous randomised trials and meta-analyses have demonstrated that statins significantly reduce the incidence of myocardial infarction, stroke, coronary revascularisation, and cardiovascular mortality in individuals at elevated cardiovascular risk [3].

Current guidelines advocate a risk-based approach to statin initiation using validated risk assessment tools, including SCORE2 and the pooled cohort equations recommended by the American College of Cardiology and the American Heart Association [4,8]. Individuals with diabetes mellitus, familial hypercholesterolaemia, chronic kidney disease, or multiple cardiovascular risk factors derive particularly substantial benefits from lipid-lowering therapy.

Evidence suggests that earlier initiation and longer exposure to LDL-C reduction confer greater cumulative benefits over the life course. Mendelian randomization studies indicate that lifelong reductions in LDL-C may provide larger cardiovascular benefits than treatment initiated later in life [6].

Nevertheless, statins remain underutilised in primary prevention, and concerns regarding adverse effects, low perceived risk, and poor adherence continue to limit their effectiveness. Shared decision-making and individualised risk communication are therefore essential to improve treatment acceptance and long-term persistence.

### 4.2. Secondary Prevention

In patients with established ASCVD, statins represent the cornerstone of secondary prevention. High-intensity statin therapy reduces recurrent cardiovascular events and cardiovascular mortality and is recommended for patients with previous myocardial infarction, ischemic stroke, or peripheral arterial disease [1].

Contemporary guidelines advocate aggressive LDL-C lowering, with target concentrations below 1.4 mmol/L (55 mg/dL) in very high-risk individuals [1]. Evidence from randomised clinical trials and meta-analyses consistently demonstrates that lower LDL-C levels are associated with progressively greater reductions in cardiovascular events, without evidence of a lower threshold beyond which benefit disappears [9].

Despite the remarkable efficacy of statins, residual cardiovascular risk persists in many patients. This observation has prompted increasing use of combination lipid-lowering therapies, including ezetimibe, PCSK9 inhibitors, and, more recently, bempedoic acid. These approaches are particularly valuable among individuals who fail to achieve LDL-C targets or who experience statin intolerance.

Importantly, treatment effectiveness depends not only on pharmacological efficacy but also on long-term adherence and persistence. Consequently, behavioural, psychosocial, and healthcare system factors have become increasingly important considerations in contemporary cardiovascular prevention.

## 5. Medication Adherence and Persistence

Adherence and long-term persistence are essential determinants of the effectiveness of statin therapy. Although statins are among the most effective preventive interventions available, approximately 40–60% of patients discontinue therapy within the first two years, thereby reducing the potential cardiovascular benefits associated with sustained LDL-C lowering [10].

Medication adherence is a multifactorial phenomenon influenced by behavioural, psychological, social, clinical, and healthcare system factors. The Necessity–Concerns Framework proposed by Horne and colleagues suggests that patients continuously balance their perceived need for medication against concerns regarding adverse effects and long-term safety [11]. Patients who perceive statins as essential for reducing cardiovascular risk are more likely to maintain treatment, whereas concerns regarding side effects frequently contribute to poor adherence.

### 5.1. Behavioural and Psychological Determinants

Fear of adverse effects, particularly statin-associated muscle symptoms (SAMSs), represents one of the most important causes of treatment discontinuation. In addition to biological mechanisms, symptom perception may be influenced by expectations, anxiety, and exposure to negative media messages. Evidence from the SAMSON and StatinWISE trials demonstrated that a substantial proportion of symptoms attributed to statins may reflect nocebo effects rather than true pharmacological toxicity [12,13].

Risk perception also influences treatment adherence. Individuals who underestimate their cardiovascular risk or who remain asymptomatic despite elevated cholesterol levels may perceive limited benefits from preventive therapy and therefore exhibit poorer persistence. Psychological conditions including anxiety, depression, and health-related worries have similarly been associated with reduced adherence and medication discontinuation.

Effective physician–patient communication, shared decision-making, and evidence-based counselling are therefore essential components of successful long-term cardiovascular prevention.

### 5.2. Healthcare System and Socioeconomic Factors

Adherence is influenced not only by individual beliefs but also by healthcare accessibility, health literacy, medication costs, and continuity of care. Inadequate follow-up, fragmented healthcare systems, and poor communication between patients and healthcare professionals may contribute to reduced treatment persistence.

Socioeconomic disadvantage may further exacerbate inequalities in cardiovascular outcomes. Limited access to healthcare services, financial barriers, and lower educational attainment are associated with poorer medication adherence and suboptimal cardiovascular risk management. These findings emphasise that treatment success depends not only on patient behaviour but also on broader structural determinants of health.

Multidisciplinary care, pharmacist-led interventions, patient education programmes, and digital technologies such as mobile reminders and telemedicine have shown promise in improving adherence, although their long-term effectiveness remains variable.

### 5.3. Social Determinants of Health and Gender Inequalities

Growing evidence suggests that social determinants of health play a critical role in cardiovascular prevention and treatment adherence. A systematic review by Costa et al. (2023) highlighted the importance of socioeconomic status, educational attainment, healthcare access, and gender inequalities in shaping vascular disease outcomes and the utilisation of preventive therapies [5].

Gender-related disparities are particularly relevant in the context of statin therapy. Women are less likely to receive guideline-recommended lipid-lowering treatment and are more likely to discontinue therapy than men. Several factors contribute to these differences, including lower perceived cardiovascular risk, concerns regarding adverse effects, and differences in healthcare-seeking behaviour [5].

Women also appear more likely to report statin-associated muscle symptoms and may exhibit greater susceptibility to negative expectations surrounding medication use. Consequently, symptom perception and nocebo effects may interact with social and gender-related factors to influence treatment adherence and persistence.

Health literacy, cultural beliefs, social support, and access to healthcare services similarly contribute to inequalities in cardiovascular prevention. These findings support the growing recognition that successful statin therapy depends not only on biological mechanisms and individual behaviour but also on broader social contexts and structural determinants of health.

Consequently, contemporary cardiovascular prevention should adopt a patient-centred and equity-oriented approach that addresses social inequalities and recognises gender-specific barriers to treatment initiation, adherence, and outcomes.

## 6. Statin-Associated Muscle Symptoms (SAMSs)

Statin-associated muscle symptoms (SAMSs) are among the most frequently reported adverse effects of statin therapy and represent one of the major barriers to long-term adherence and treatment persistence. SAMSs encompass a spectrum of muscle-related complaints, including myalgia, muscle weakness, cramps, and fatigue, which may occur with or without biochemical evidence of muscle injury [14]. Although most patients tolerate statins well, concerns regarding muscle symptoms frequently contribute to dose reduction, treatment discontinuation, and failure to achieve optimal cardiovascular risk reduction.

Importantly, the terms “statin-associated muscle symptoms”, “statin intolerance”, and “statin-induced myopathy” should not be used interchangeably. These entities differ in their pathophysiology, clinical presentation, and diagnostic certainty.

### 6.1. True Statin-Induced Myopathy

True statin-induced myopathy is uncommon and is characterised by objective evidence of skeletal muscle injury. Clinical manifestations range from mild elevations in creatine kinase (CK) to severe myonecrosis and, rarely, rhabdomyolysis. The incidence of clinically significant myopathy is estimated to be less than 0.1% among patients receiving statin therapy [14].

Several biological mechanisms have been proposed to explain statin-related muscle injury, including mitochondrial dysfunction, impaired oxidative phosphorylation, depletion of coenzyme Q10, disturbances in calcium homeostasis, oxidative stress, and genetic susceptibility. Variants in genes involved in statin metabolism and transport, particularly SLCO1B1 polymorphisms, have been associated with an increased risk of myopathy [15].

Despite these mechanistic insights, establishing a direct causal relationship between statin exposure and muscle dysfunction remains challenging because muscle symptoms are common in ageing populations and may arise from multiple coexisting conditions, including physical inactivity, sarcopenia, hypothyroidism, vitamin D deficiency, and rheumatological disorders.

### 6.2. Statin Intolerance

Statin intolerance represents a broader clinical syndrome characterised by the inability to tolerate the dose intensity required to achieve therapeutic LDL-C targets owing to adverse effects that improve following dose reduction or discontinuation and recur upon rechallenge. According to the National Lipid Association, complete statin intolerance is uncommon, whereas partial intolerance, in which patients tolerate lower doses or alternative regimens, is more frequent [14].

The diagnosis of statin intolerance requires careful evaluation because many symptoms attributed to statins are nonspecific and may reflect underlying comorbidities or age-related musculoskeletal conditions. Consequently, clinicians should exclude alternative causes of muscle symptoms before attributing them solely to statin therapy.

Recognition of partial intolerance is particularly important because many patients can successfully continue treatment through dose modification, alternate-day dosing, switching to a different statin, or the use of combination lipid-lowering therapy.

### 6.3. Nocebo Effect and Psychological Contributors

Increasing evidence suggests that psychological and contextual factors contribute to symptom perception among individuals receiving statins. Negative expectations regarding treatment, fear of adverse effects, health anxiety, and exposure to misinformation may amplify symptom reporting through nocebo mechanisms.

The SAMSON trial demonstrated that approximately 90% of the symptom burden experienced during statin therapy was also observed during placebo administration, suggesting that expectations play a significant role in symptom perception [12]. Similarly, the StatinWISE trial found no significant differences in muscle symptoms between statin and placebo periods among individuals with previous statin intolerance [16].

These findings support the biopsychosocial model of SAMSs, whereby biological mechanisms interact with psychological and contextual influences to determine symptom severity and treatment behaviour. Media reports, prior experiences, physician communication, and negative beliefs about medications may all contribute to symptom development and premature discontinuation.

Nevertheless, recognition of nocebo effects should not lead clinicians to dismiss patient concerns. Instead, effective communication and shared decision-making are required to address fears while maintaining therapeutic trust.

### 6.4. Clinical Diagnosis and Differential Diagnosis

Diagnosis of SAMSs remains clinical because no definitive biomarker currently exists. Diagnosis relies on several factors, including the following:

- The temporal association between symptom onset and statin initiation;
- Improvement following treatment interruption;
- Recurrence after rechallenge;
- Dose–response relationships;
- The exclusion of alternative causes of muscle symptoms.

Measurement of creatine kinase may assist in identifying severe muscle injury, although normal CK levels do not exclude SAMSs. Additional investigations should consider thyroid dysfunction, vitamin D deficiency, inflammatory myopathies, renal impairment, drug interactions, and age-related sarcopenia.

Because musculoskeletal complaints are highly prevalent among older adults, distinguishing true statin-related symptoms from coincidental symptoms remains one of the greatest challenges in clinical practice.

### 6.5. Clinical Management of SAMS

Management strategies should aim to maintain adequate cardiovascular protection while minimising symptom burden. Premature discontinuation of statins should be avoided because interruption of therapy is associated with increased cardiovascular morbidity and mortality.

Recommended approaches include the following:

- Reducing statin dose intensity;
- Switching to an alternative statin;
- Intermittent or alternate-day dosing;
- Addressing reversible causes of symptoms;
- Correcting hypothyroidism or vitamin D deficiency;
- Lifestyle modification and exercise interventions;
- Implementation of shared decision-making and patient education.

Many patients previously considered statin-intolerant are able to tolerate lower doses or alternative statins, emphasising the importance of individualised treatment strategies.

### 6.6. Combination Lipid-Lowering Therapy and Alternatives to Statins

The increasing recognition of statin intolerance has stimulated growing interest in non-statin lipid-lowering therapies, with contemporary cardiovascular prevention increasingly relying on combination approaches rather than statin monotherapy. Ezetimibe, which inhibits intestinal cholesterol absorption, is recommended as the first add-on therapy in patients who fail to achieve LDL-C targets with statins alone or who experience partial statin intolerance. The IMPROVE-IT trial demonstrated additional cardiovascular benefits when ezetimibe was combined with statin therapy [17]. More potent LDL-C reduction can be achieved with monoclonal antibodies targeting proprotein convertase subtilisin/kexin type 9 (PCSK9), including evolocumab and alirocumab, which significantly reduce LDL-C concentrations and cardiovascular events among high-risk individuals. The FOURIER and ODYSSEY OUTCOMES trials demonstrated the efficacy and safety of these agents in secondary prevention populations [9,18], although their widespread use remains limited by cost and accessibility. Bempedoic acid, an adenosine triphosphate citrate lyase inhibitor, lowers LDL-C through a mechanism upstream of HMG-CoA reductase. As its activation occurs in the liver rather than skeletal muscle, it may represent an attractive option for patients with statin intolerance. The CLEAR Outcomes trial demonstrated significant reductions in major adverse cardiovascular events among statin-intolerant patients receiving bempedoic acid [19]. Inclisiran, a small interfering RNA (siRNA)-based therapy, inhibits hepatic synthesis of PCSK9 and provides prolonged LDL-C reduction with twice-yearly dosing. This approach may improve adherence and simplify long-term lipid management. Although clinical trials have demonstrated substantial LDL-C reductions, long-term cardiovascular outcome data remain limited, and its real-world effectiveness requires further evaluation.

## 7. Muscle Function and Physiological Outcomes

Traditional evaluation of statin therapy has predominantly focused on its ability to lower LDL-C and reduce adverse cardiovascular events. These outcomes remain central to determining the clinical efficacy of statins; however, increasing attention is being directed towards broader physiological measures, including muscle strength, skeletal muscle mass, physical performance, and cellular health.

This growing interest reflects the recognition that cardiovascular health extends beyond the prevention of major cardiovascular events and includes the preservation of functional capacity, independence, and overall physiological reserve. Measures of muscle function and body composition have been shown to be important predictors of frailty, functional dependence, reduced quality of life, and mortality, particularly among older individuals and patients with multimorbidity. Their assessment may therefore provide additional information regarding a patient’s overall health and functional status.

The relevance of these physiological measures to statin therapy is particularly important in the context of statin-associated muscle symptoms, where patients frequently report muscle pain, weakness, or reduced physical capacity. However, it remains unclear whether measurable changes in muscle strength, skeletal muscle mass, or cellular health are directly attributable to statin therapy or instead reflect ageing, cardiovascular disease, comorbid conditions, physical inactivity, and other factors that influence muscle function.

Contemporary evidence must therefore be interpreted carefully to distinguish established indicators of general physiological health from emerging measures that may potentially contribute to the objective assessment of statin-related muscle dysfunction.

### 7.1. Muscle Function and Physiological Outcomes in Statin Therapy

Muscle strength, physical performance, and body composition are increasingly recognised as important indicators of overall health and predictors of morbidity and mortality across diverse populations. Handgrip strength is widely regarded as a robust measure of muscle function and physiological reserve, with low handgrip strength associated with increased risks of frailty, disability, cardiovascular disease, hospitalisation, and all-cause mortality [20]. Similarly, preservation of skeletal muscle mass is important for healthy ageing, as reduced muscle mass is associated with sarcopenia, impaired mobility, exercise intolerance, falls, and reduced quality of life. These relationships have been demonstrated in observational studies involving older individuals and patients with cardiovascular disease. Phase angle, derived from bioelectrical impedance analysis, has also emerged as a physiological marker reflecting cellular membrane integrity, nutritional status, and physiological reserve. Lower phase angle values have been associated with chronic inflammation, frailty, poor nutritional status, and adverse outcomes in several chronic diseases, including cardiovascular disease [21]. Despite the prognostic value of these parameters as indicators of overall health, there is currently insufficient high-quality evidence to support their use as specific biomarkers of statin-related muscle toxicity.

Recent research has therefore explored whether objective physiological measures may improve the assessment of statin-associated muscle symptoms and identify possible statin-related muscle dysfunction. Observational studies investigating the relationship between muscle strength and statin exposure have reported inconsistent findings, with some suggesting modest reductions in muscle strength among individuals experiencing muscle symptoms. However, these associations are frequently influenced by age, physical inactivity, multimorbidity, obesity, and pre-existing sarcopenia, making it difficult to establish an independent effect of statin therapy on muscle strength. Similar concerns apply to skeletal muscle mass. Although experimental studies have proposed biological mechanisms through which statins may contribute to muscle loss, consistent reductions in skeletal muscle mass attributable to statin therapy have not been demonstrated in clinical settings.

Reduced muscle mass is more commonly associated with ageing, chronic disease, physical inactivity, and exercise avoidance secondary to muscle symptoms. Interest has also grown in the use of phase angle as a marker of cellular health in individuals receiving statin therapy. Although lower phase angle values are associated with adverse outcomes in several chronic diseases, few studies have specifically examined the relationship between phase angle and statin exposure, and there are currently no validated thresholds for the use of bioelectrical impedance analysis to identify statin-induced muscle dysfunction. Emerging technologies, including wearable devices and remote monitoring of physical activity, may further contribute to the objective assessment of physiological function in patients receiving statins; however, evidence remains insufficient to support their routine clinical implementation. Therefore, handgrip strength, skeletal muscle mass, and phase angle should currently be regarded primarily as markers of overall physiological function and reserve rather than specific indicators of statin toxicity, and their clinical value may lie in complementing traditional assessments and patient-reported symptoms.

### 7.2. Areas of Controversy

An important area of uncertainty is whether reductions in muscle function observed among statin-treated individuals are directly attributable to statin therapy or reflect the effects of ageing, frailty, chronic disease, and physical inactivity. Statins are frequently prescribed to older individuals with multimorbidity and established cardiovascular risk factors, many of whom already have conditions associated with impaired muscle function [14,20]. These overlapping factors make it difficult to isolate the independent effect of statin exposure on physiological outcomes. Furthermore, the available evidence remains heterogeneous, and adequately designed prospective studies capable of distinguishing statin-related changes from underlying age- and disease-related decline are limited [14].

The interpretation of patient-reported muscle symptoms presents an additional challenge. Evidence from placebo-controlled and N-of-1 trials has demonstrated that symptoms attributed to statin therapy may also occur during placebo exposure, highlighting the influence of symptom perception and nocebo mechanisms [12,16]. Individuals experiencing muscle symptoms may also reduce their level of physical activity because of pain, perceived weakness, or concerns regarding symptom exacerbation. Consequently, reductions in muscle strength or physical performance may partly reflect secondary behavioural changes rather than a direct pharmacological effect of statins. This interaction between symptom perception, physical activity, and physiological function further complicates attempts to establish causality.

Considerable uncertainty also surrounds the clinical application of emerging physiological markers. Although measures such as handgrip strength, skeletal muscle mass, gait speed, and phase angle have prognostic value as indicators of general health and physiological reserve, none currently demonstrate sufficient sensitivity or specificity to identify statin-induced muscle injury [14,20,21]. Their use as diagnostic tools for statin-associated muscle symptoms therefore remains investigational and requires further prospective validation.

Finally, it remains uncertain whether the routine assessment or improvement of physiological parameters should represent a specific therapeutic target in cardiovascular prevention. Preservation of muscle function is clearly important for healthy ageing, functional independence, and quality of life [20]; however, evidence that routine measurement of these parameters improves cardiovascular outcomes or meaningfully guides decisions regarding statin therapy remains limited. These uncertainties highlight the need for carefully designed longitudinal studies integrating physiological measures with clinical assessment, patient-reported symptoms, and biological markers.

### 7.3. Remaining Knowledge Gaps and Future Research

Several important knowledge gaps remain before physiological measures can be incorporated into the routine assessment of patients receiving statin therapy. A major limitation of the current evidence is the difficulty in distinguishing statin-related muscle dysfunction from changes associated with ageing, cardiovascular disease, multimorbidity, sarcopenia, and physical inactivity [14,20]. Large prospective longitudinal studies are therefore required to determine whether objective measures of muscle strength, body composition, and cellular health can identify changes specifically associated with statin exposure or statin-associated muscle symptoms.

Future research should also evaluate whether combining physiological measures with molecular biomarkers, pharmacogenomic data, and patient-reported outcomes improves the assessment of SAMSs. Given the heterogeneous and multifactorial nature of muscle symptoms, a multidimensional approach may provide greater clinical value than reliance on a single biomarker [14,15]. However, such models require prospective validation and assessment of their diagnostic performance before routine clinical implementation.

Digital health technologies and wearable sensors may provide additional opportunities for longitudinal assessment of physical activity, functional capacity, and symptom progression in real-world settings [22]. Continuous or repeated measurements could potentially identify temporal relationships between statin exposure, symptom development, and changes in physical function. Nevertheless, the feasibility, accuracy, clinical relevance, and cost-effectiveness of these approaches remain uncertain and require further investigation [23].

A further limitation of the existing evidence is the underrepresentation of ethnically diverse populations and individuals from low- and middle-income countries. Greater inclusion of these populations is necessary to establish whether emerging physiological assessment strategies are generalisable across different demographic groups and healthcare settings. Current evidence therefore supports handgrip strength, skeletal muscle mass, and phase angle as indicators of overall physiological and functional status [20,21], but not as validated biomarkers of statin-related muscle toxicity [14]. Their potential value may lie within a multidimensional assessment framework that integrates physiological, biological, behavioural, and clinical information to support more individualised cardiovascular care.

## 8. Contemporary Guidelines and Precision Cardiovascular Prevention

Contemporary cardiovascular prevention has evolved beyond a uniform approach toward increasingly individualised strategies that incorporate clinical characteristics, genetic information, behavioural factors, and technological innovations. Current guidelines from the European Society of Cardiology (ESC) and the American College of Cardiology/American Heart Association (ACC/AHA) emphasise personalised risk assessment and shared decision-making to optimise lipid-lowering therapy and improve long-term cardiovascular outcomes [1,4].

Although these developments have generated considerable enthusiasm, the translation of precision medicine into routine clinical practice remains challenging. Issues relating to accessibility, cost-effectiveness, external validity, and health inequalities continue to limit widespread implementation.

### 8.1. Individualised Cardiovascular Risk Assessment

Current guidelines recommend that treatment decisions should be guided by absolute cardiovascular risk rather than isolated cholesterol concentrations. Risk prediction models such as SCORE2 and the pooled cohort equations provide a framework for estimating cardiovascular risk and identifying individuals most likely to benefit from intensive lipid-lowering interventions [8].

In addition to traditional risk factors, contemporary approaches increasingly incorporate risk-enhancing conditions, including family history, chronic inflammatory disorders, chronic kidney disease, diabetes mellitus, and biomarkers such as lipoprotein(a). Coronary artery calcium scoring has further improved risk stratification, particularly among individuals with intermediate cardiovascular risk.

Nevertheless, risk prediction models have important limitations. Many algorithms were developed using European and North American populations and may not perform optimally in ethnically diverse or low-resource settings. Consequently, calibration and validation within underrepresented populations remain important priorities.

### 8.2. Pharmacogenomics and Polygenic Risk Scores

Pharmacogenomics has emerged as a promising approach for improving the safety and effectiveness of statin therapy. Genetic variants influencing statin metabolism and transport, particularly polymorphisms in the SLCO1B1 gene, have been associated with increased susceptibility to statin-associated muscle symptoms and may facilitate individualised treatment selection [15].

Similarly, polygenic risk scores (PRSs) have attracted considerable interest as tools for identifying individuals at increased genetic risk of atherosclerotic cardiovascular disease. Some studies suggest that individuals with elevated genetic risk derive greater absolute benefits from LDL-C lowering and intensive preventive interventions.

Despite these advances, the clinical application of pharmacogenomics and polygenic risk scores remains limited. Several challenges persist, including the following:

- Insufficient prospective validation;
- Uncertainty regarding cost-effectiveness;
- A lack of consensus regarding interpretation and clinical thresholds;
- Limited availability in routine healthcare settings;
- Concerns regarding ethical and privacy issues;
- Inadequate representation of diverse ancestral populations.

Importantly, most genomic studies have been conducted in populations of European ancestry. African populations remain underrepresented in genomic databases, raising concerns regarding the transferability and accuracy of genetic risk prediction across different ethnic groups. Consequently, wider implementation of genomic medicine will require greater inclusivity and validation in real-world populations.

### 8.3. Shared Decision-Making and Patient-Centred Care

Recognition of the importance of patient preferences has shifted cardiovascular prevention toward shared decision-making. This approach acknowledges that treatment adherence is influenced not only by clinical risk but also by patients’ beliefs, expectations, concerns, and social circumstances.

Shared decision-making may improve adherence, increase treatment satisfaction, and reduce misconceptions surrounding statin therapy. Effective communication is particularly important in addressing fears regarding adverse effects and mitigating nocebo responses.

However, implementation of patient-centred care presents practical challenges. Time constraints, limited health literacy, cultural differences, and inadequate communication skills may hinder meaningful engagement between healthcare professionals and patients. Furthermore, disparities in healthcare access and educational attainment may prevent some populations from fully benefiting from shared decision-making strategies.

### 8.4. Digital Health Technologies

Rapid advances in digital health have created new opportunities for improving cardiovascular prevention. Mobile health applications, wearable devices, telemedicine platforms, and electronic reminders have been used to promote medication adherence, facilitate lifestyle modification, and enhance patient monitoring.

Digital technologies may provide several advantages, including the following:

- Improved continuity of care;
- Enhanced patient engagement;
- Remote monitoring capabilities;
- Personalised behavioural interventions;
- Greater accessibility for geographically isolated populations.

Nevertheless, evidence supporting sustained long-term benefits remains inconsistent. Digital interventions frequently demonstrate declining effectiveness over time, and issues related to digital literacy, user engagement, data security, and privacy continue to present significant challenges.

Moreover, the digital divide represents an important barrier, particularly in low- and middle-income countries where access to technology and internet infrastructure may be limited. Consequently, digital health should be regarded as a complementary strategy rather than a substitute for conventional clinical care.

### 8.5. Artificial Intelligence and Predictive Analytics

Artificial intelligence (AI) and machine learning have emerged as powerful tools for cardiovascular risk prediction, imaging analysis, and treatment optimisation. Machine learning algorithms have demonstrated promising performance in identifying complex interactions among clinical variables and predicting cardiovascular events beyond traditional statistical approaches.

Potential applications include the following:

- Individualised cardiovascular risk prediction;
- Prediction of medication adherence;
- Identification of patients at risk of statin intolerance;
- Automated interpretation of imaging data;
- The development of personalised preventive strategies.

Despite these advances, several limitations constrain clinical implementation. Many AI models have been developed using retrospective datasets and lack external validation. Algorithmic bias, overfitting, and limited interpretability remain important concerns. Furthermore, most machine learning studies involve populations from high-income countries, raising questions regarding their applicability to diverse healthcare settings.

Real-world evidence supporting the impact of AI on clinical outcomes remains limited, and further prospective studies are required before widespread implementation can be recommended.

### 8.6. Accessibility and Equity of Precision Cardiovascular Medicine

Although precision medicine offers considerable promise, economic considerations and accessibility remain central to its implementation. Genetic testing, advanced imaging, digital health technologies, and novel lipid-lowering therapies may increase healthcare costs and are often inaccessible in resource-constrained settings. Cost-effectiveness analyses suggest that the benefits of precision approaches vary according to healthcare systems, population characteristics, and baseline cardiovascular risk. Consequently, widespread adoption of these technologies may exacerbate existing health inequalities if access remains restricted to affluent populations. These concerns are particularly relevant in low- and middle-income countries, where competing healthcare priorities and limited resources necessitate careful allocation of healthcare expenditure. Furthermore, most evidence underpinning precision cardiovascular medicine originates from studies conducted in Europe and North America, with African populations remaining markedly underrepresented in clinical trials, genomic studies, and machine learning datasets. This lack of representation limits the generalisability of risk prediction models, pharmacogenomic algorithms, and polygenic risk scores, particularly as variations in genetic architecture, environmental exposures, and healthcare systems may influence both cardiovascular risk and treatment responses. Addressing these disparities will require increased investment in African cardiovascular research, the development of region-specific datasets, and greater inclusion of diverse populations in international clinical studies. Ensuring that precision cardiovascular medicine is both accessible and representative will be essential to prevent technological advances from further widening existing health inequalities.

## 9. Future Directions

Despite decades of clinical experience and a substantial body of evidence supporting statin therapy, several important scientific and clinical questions remain unresolved. Future research should focus not only on optimising lipid lowering but also on improving adherence, understanding the complex nature of statin-associated symptoms, and facilitating the equitable implementation of precision cardiovascular medicine. Although the cardiovascular efficacy of statins is well established, the pathophysiology and detection of statin-associated muscle symptoms remain controversial. Differentiating symptoms directly attributable to statin therapy from those related to ageing, comorbid disease, physical inactivity, or the nocebo effect remains challenging, and the relative contribution of biological and psychological mechanisms to symptom development is still poorly understood.

The absence of reliable biomarkers capable of objectively identifying statin-associated muscle injury represents a major limitation in current clinical practice. Novel physiological and genetic approaches, including handgrip strength measurement, assessment of skeletal muscle mass and phase angle, pharmacogenomics, and polygenic risk scores, have shown promise; however, their clinical relevance and ability to improve patient outcomes require further investigation. Emerging technologies involving metabolomics, proteomics, transcriptomics, and multi-omics approaches may provide additional insight into the biological mechanisms underlying statin intolerance. These methods remain largely investigational and require prospective validation before routine clinical implementation. Similarly, further research is needed to establish whether measures such as handgrip strength, skeletal muscle mass, and phase angle provide clinically meaningful information beyond traditional cardiovascular risk factors and patient-reported outcomes.

Suboptimal adherence remains one of the greatest barriers to maximising the cardiovascular benefits of statin therapy. Future interventions should address the multifactorial determinants of treatment persistence by integrating behavioural, psychological, and healthcare system perspectives. Patient education, shared decision-making, pharmacist-led interventions, digital reminders, telemedicine, and multidisciplinary models of care have demonstrated varying degrees of effectiveness, although evidence regarding their long-term sustainability and cost-effectiveness remains limited. Greater emphasis should also be placed on addressing misinformation, improving health literacy, and developing strategies to reduce nocebo responses while preserving trust between patients and healthcare professionals.

Traditional approaches to cardiovascular prevention have predominantly focused on biological risk factors; however, increasing evidence suggests that successful long-term prevention requires the integration of biological, behavioural, psychological, and social determinants of health. Future conceptual models should therefore adopt a biopsychosocial framework that recognises interactions between genetic susceptibility, symptom perception, health beliefs, treatment expectations, healthcare access, and broader social influences. Such approaches may facilitate more individualised and patient-centred interventions. Importantly, the management of statin-associated muscle symptoms should move beyond simplistic explanations based solely on pharmacological toxicity and acknowledge the complex interplay between biological and contextual factors.

A further priority is the inclusion of more diverse populations in cardiovascular research. Populations from low- and middle-income countries, particularly those in Africa, remain underrepresented in clinical trials, genomic studies, and the development of machine learning models. The predominance of populations of European ancestry in existing research limits the generalisability of findings across diverse healthcare settings. Future studies should prioritise the inclusion of ethnically diverse populations, the development of region-specific risk prediction models, expansion of genomic databases involving African populations, generation of real-world evidence from low-resource settings, and evaluation of implementation strategies across different healthcare systems. Addressing these gaps will be essential to ensure that advances in cardiovascular prevention do not exacerbate existing health inequalities.

Precision cardiovascular medicine offers considerable promise; however, substantial barriers to implementation remain, including cost, accessibility, infrastructure requirements, digital literacy, ethical considerations, and limited external validation. Future studies should focus on prospective validation, cost-effectiveness analyses, and implementation science to determine whether emerging technologies translate into improved clinical outcomes in real-world settings. Particular attention should be given to ensuring equitable access to innovation and preventing further disparities in healthcare delivery. Ultimately, the success of precision cardiovascular medicine will depend not only on technological advancement but also on its affordability, accessibility, and ability to address the needs of diverse populations.

## 10. Conclusions

Statins remain the cornerstone of lipid-lowering therapy, supported by robust evidence demonstrating substantial reductions in cardiovascular morbidity and mortality across both primary and secondary prevention settings. The causal relationship between LDL-C reduction and atherosclerotic cardiovascular risk is well established, and sustained lipid lowering remains fundamental to contemporary cardiovascular prevention. However, the effectiveness of statin therapy in clinical practice is determined not only by pharmacological efficacy but also by treatment adherence, tolerability, medication beliefs, symptom perception, and broader social and healthcare system factors.

Current evidence increasingly supports a multifactorial interpretation of statin-associated muscle symptoms, in which biological mechanisms may interact with psychological, behavioural, and contextual influences. Although emerging physiological measures, including muscle strength, skeletal muscle mass, and phase angle, provide valuable information regarding overall functional and physiological status, evidence remains insufficient to support their use as specific biomarkers of statin-related muscle toxicity. Similarly, pharmacogenomics, polygenic risk scores, artificial intelligence, and digital health technologies offer opportunities to individualise cardiovascular prevention, but their clinical utility, cost-effectiveness, external validity, and accessibility require further prospective evaluation.

Contemporary cardiovascular prevention should therefore integrate evidence-based lipid lowering with individualised risk assessment, shared decision-making, and strategies that address adherence and patient perceptions. Precision cardiovascular medicine represents an important future direction, but its successful implementation will depend on robust clinical validation and equitable access across diverse populations and healthcare systems. Future research should prioritise the development of objective approaches to evaluating statin-associated symptoms, improved understanding of the determinants of long-term treatment persistence, and greater representation of populations from low- and middle-income countries. Integrating biological, physiological, behavioural, and social evidence may ultimately provide a more comprehensive and patient-centred approach to cardiovascular risk reduction.

## Data Availability

The original contributions presented in this study are included in the article/Supplementary Materials. Further inquiries can be directed to the corresponding author(s).

## Supplementary Materials

The following supporting information can be downloaded at: https://www.mdpi.com/article/10.3390/ijerph23070921/s1, Table S1: Complete PubMed Search Strategy.
