# Supplementary material for: Statin Therapy and Cardiovascular Prevention: Contemporary Evidence, Challenges, and Future Directions—A Narrative Review
Source: Int J Environ Res Public Health. 2026 Jul 17;23(7):921. doi: 10.3390/ijerph23070921 (PMC13410502; doi:10.3390/ijerph23070921)
Supplement: Supplementary file 1 [file ijerph-23-00921-s001.zip › ijerph-4407848-supplementary.pdf]

Supplementary Table S1. Complete PubMed Search Strategy

("Hydroxymethylglutaryl-CoA Reductase Inhibitors"[Mesh] OR statin* OR atorvastatin OR rosuvastatin OR simvastatin OR pravastatin OR pitavastatin)

AND

("Cardiovascular Diseases/prevention and control"[Mesh] OR "cardiovascular prevention" OR ASCVD OR "atherosclerotic cardiovascular disease")

AND

("Medication Adherence"[Mesh] OR adherence OR persistence OR compliance

OR "Statin-Associated Muscle Symptoms"

OR "statin intolerance"

OR nocebo

OR "muscle strength"

OR "handgrip strength"

OR "skeletal muscle"

OR pharmacogenomics

OR "precision medicine"

OR "artificial intelligence")

Filters:

English; Humans; January 2015–March 2026.
